# Defining early health technology assessment: reclaiming origins and diversity of applications

**DOI:** 10.1017/S0266462325100159

**Published:** 2025-06-02

**Authors:** Clifford Goodman

**Affiliations:** Independent Consultant, Health Care Technology & Policy, Bethesda, MD, USA

**Keywords:** technology assessment, biomedical, diffusion of innovation, health policy

## Introduction

The article on defining early health technology assessment (eHTA) by Grutters et al. (1) of the working group under Health Technology Assessment international (HTAi) is most welcome for its comprehensive approach, timeliness, and scope, all of which are inherent in the definition itself:

a health technology assessment conducted to inform decisions about subsequent development, research and/or investment by explicitly evaluating the potential value of a conceptual or actual health technology.

Whether or not envisioned by the working group, the definition also reflects core aspects of the field’s origin. Leading up to the emergence and formal recognition of *health* technology assessment, the origins of *technology assessment* in the 1960s and early 1970s in the United States emphasized early and open-ended revisits along the technology life cycle:

[Technology assessment is] the systematic study of the effects on society, that may occur when a technology is introduced, extended, or modified, with emphasis on the impacts that are unintended, indirect, or delayed (2).

Toward informing “subsequent development, research, and/or investment,” eHTA is inherently a “look-ahead” endeavor, starting as early as a “conceptual” health technology. Furthermore, as the definition suggests and the article conveys, the scope of “potential value” is open to probing a wider set of stakeholder-relevant impacts and related considerations. These would include “unintended, indirect, or delayed” impacts. Factors and events – ranging from an increasing focus on patient-centered dimensions of value to certain worsening social, economic, and health disparities, as well as environmental and sustainability concerns – remind us to break out of a constrained vision of HTA. The future orientation and wide impact aperture for HTA are not original, but they have been de-emphasized or crowded out of our field in the last three decades.

Among the various fields in which technology assessment has been applied, HTA has taken on a disproportionately large volume, though a more narrowly focused scope of assessed impacts. A detailed thematic analysis of publications indexed in the Scopus database of peer-reviewed literature across all disciplines (including sciences, technology, engineering, economics, mathematics, etc.) with the search term “technology assessment” during the years 1969–2015 revealed that nearly 70 percent were in the fields of medicine and health-related sciences. Mindful of the field’s origins, the investigator of this analysis went on to observe of the technology assessments in medicine and health that:

[I]t is worth noticing that Health Technology Assessment is very often far in its nature from a participative and deliberative process involving various groups of stakeholders and providing space for group reflection on the possible long term effects of a particular medical technology. It is rather a very rigid scientific (clinical tests) and administrative (projection of the increase/decrease of public spending) process aiming at obtaining relevant permissions from authorities to introduce a particular technology into medical practice (3).

In the decade since the timeframe of that analysis, the field of HTA has increasingly developed and cited aspects of best practice, including stakeholder engagement, patient and citizen involvement, and the expansion of the dimensions of “value” beyond safety, efficacy/effectiveness, cost-effectiveness, and budget impact. Nevertheless, recognizing that the conduct of HTA by designated bodies is typically delimited by their respective mandates or other remits, the general observation of a confined scope of HTA remains fair. Consider, then, the wider scope of potential interests of eHTA and diverse instances of how eHTA might inform different types of decisions about further development, research, and investment in health technology.

## Who conducts eHTA?

The scope of the definition makes clear that eHTA is not confined to HTA agencies. Consider the main roles or purposes of HTA today, some of which are not formally labeled as HTA:

- Advise payers (health authorities, health plans, etc.) about technology coverage and payment
- Advise/guide clinicians and patients about technology use (e.g., with evidence-based clinical practice guidelines)
- Help managers of hospitals, health care networks, and other provider institutions make decisions about acquiring or investing in technology
- Support decisions by national and regional public health authorities about conducting population health programs
- Support decisions by health technology companies about technology development and marketing
- Support decisions by investors in the health care sector
- Inform research agencies about unmet health care needs and evidence gaps

In each case, a form of HTA can be conducted during the developmental, premarket stage of a technology or class of technologies, followed at the time of market entry or subsequently as the need arises for further decision support. What forms can eHTA take across these roles of HTA? As demonstrative examples, consider how eHTA can be conducted from these three perspectives: a biotechnology investor, a hospital network, and a novel therapy manufacturer.

## Biotechnology investor: toward an exit

A biotech investor could conduct a form of eHTA focusing on the ability of a developmental technology to achieve milestones or “proof points” that would trigger or enable a high-return exit strategy, whether via a merger or acquisition, initial public offering, licensing agreement, or strategic partnership. Chief among these milestones would be, for example, regulatory approval to conduct clinical trials; phase I or II clinical trial results; regulatory “wins,” that is, clearances or market approvals; and nontrial, real-world evidence in heterogeneous patient populations. Other milestones or pivotal decision points might include regulatory agency designation for “breakthrough” status or an accelerated approval pathway based on the demonstration of unmet medical needs or early evidence suggesting substantial clinical benefit. Further key factors weighed in eHTA might include the anticipated rollout of clinical indications and their respective target population sizes, installing a proven management team, partnership announcements, and the outlook for stable supply chains and manufacturing capacity.

Failure to achieve primary endpoints, evidence of toxicity or adverse events, regulatory uncertainties (delays, hurdles, or understaffed agencies), ambiguities in intellectual property, and being “leap-frogged” by competing technologies are among the factors that can plunge value and derail successful exit strategies. Of course, broader economic and political conditions can affect entire markets for emerging health care technologies, including inflation, pricing, international trade hurdles, access to leading-edge expertise, and political instability.

## Hospital network information technology acquisition

Consider a hospital network conducting eHTA for investing in a next-generation artificial intelligence (AI) platform to manage its information technology (IT) operations. This likely would occur in a context of steeply increasing AI capabilities, including, for example, the prospect of multiple interacting AI agents coordinating diverse IT operations across the hospital network. An eHTA might require examination of an extensive, open set of impacts that could be grouped under such categories as strategic alignment, patient impacts, economic impacts, efficiency, sustainability, governance, and legal/regulatory compliance, as shown in Table 1.

**Table 1.** Potential types of impact for eHTA of an AI platform for hospital network IT operations

*Strategic alignment* Pursuant to hospital’s vision or strategic plan Competitive advantage, e.g., maintain or grow market share
*Patient impacts* Patient safety Patient outcomes Clinical guideline or pathway adherence Patient satisfaction Patient engagement (via digital tools, personalized health care) Privacy and confidentiality Patient equity
*Economic impacts* Capital budgeting Other costs (licensing, installation, systems integration, training, security, etc.) Expenditure management Revenue cycle management Data monetization (e.g., selling de-identified patient data to industry)
*Efficiency* Clinical workflow Administrative workflow Delivery site reconfiguration Workforce EHR system integration
*Sustainability* Energy consumption Maintenance and services Upgrades Consumables Supply chain
*Governance and legal/regulatory compliance* Product safety Premarket and postmarket device software regulations Patient data rights/protections Cybersecurity Transparency Deceptive/manipulative techniques Algorithmic bias/discrimination Labor protections Environmental

AI, artificial intelligence; EHR, electronic health records; eHTA, early health technology assessment; IT, information technology.

The rate of change in the expansive environment of AI in health care suggests that eHTA would have to be inclusive of stakeholders, iterative, and adaptable to inform objectively the hospital network decision-makers.

## Novel therapy manufacturer: mapping the HTA terrain

For companies active in multiple national markets, eHTA can involve “mapping” the anticipated global or regional terrain of payers and the HTA agencies that inform or otherwise influence coverage, payment, and related policies for access. This form of eHTA entails anticipating and understanding evidence expectations and related requirements across different national and regional HTA bodies. This information can inform not only the further development of a technology or a class of technologies but also the priorities for compiling evidence and the sequences and timelines for pursuing access in various national markets.

Health technology companies, including those in the pharmaceutical, biologics, device, and diagnostics industries, have become increasingly knowledgeable and active stakeholders in HTA. Companies recognize that, in most national markets, the roles of regulation and assessment of health care technologies originated and evolved separately, including regarding their respective evidence requirements. While there has been progress over the last two decades in the alignment of their evidence requirements, considerable gaps remain. Of great importance to companies for planning technology development and research is understanding and anticipating how evidence generated for regulatory approval in each country influences what additional evidence may be needed for HTA in that country. This may entail, for example, clinical trials or other prospective data on additional clinical endpoints versus a standard of care or in more heterogeneous patient groups, and longer-term real-world data on effectiveness and adverse events.

Companies also come to understand that, from country to country, HTA bodies have different remits, relationships with payers and other decision-makers, processes, methods, evidence expectations, resources, and more. The responsibilities for evaluating clinical, economic, or other impacts of health care technology can be distributed differently, including among agencies or other organizations that are not labeled as HTA bodies, and typically vary for pharmaceuticals and biologics, devices and equipment, and diagnostics. As such, eHTA has to operate within a complex, heterogeneous, and interrelated sector of evidence generation. How does a health care technology company proceed with this multinational/regional eHTA?

Among the many factors informing the strategy for further development, research, and rollout of a new technology in various countries are their respective market sizes, regulatory pathways, and the nature of HTA bodies’ influence on access. For example, the first target markets for an emerging cancer therapy with a companion diagnostic might include the European “Big 5,” that is, Germany, the United Kingdom, France, Italy, and Spain, plus the United States, China, and Japan. In addition to the evidence and related requirements of their applicable regulatory agencies, a company would examine the roles and requirements of their respective HTA agencies or related HTA-like functions of other organizations.

Of great strategic importance are differences in how HTA is conducted across and sometimes within countries (e.g., in the United States). Companies are especially interested in HTA agencies operating in their target markets as well as those that are regarded as setting examples for best practices, early adopters of alternative methods, and otherwise those influential in the field of HTA. Regarding clinical evidence expectations, companies want to know to what extent HTA bodies will require evidence beyond what will be required for regulatory approval, as suggested above.

In this instance, the company with the emerging cancer therapy and companion diagnostic might focus initially on:

- Germany: Federal Joint Committee (G-BA) and Institute for Quality and Efficiency in Health Care (IQWiG)
- England: National Institute for Health and Care Excellence (NICE)
- France: National Authority for Health (HAS)
- Italy: Italian Medicines Agency (AIFA)
- Spain: Spanish Agency of Medicines and Medical Devices (AEMPS) and Spanish Network of Agencies for Assessing National Health System Technologies and Performance (RedETS)
- US: Institute for Clinical and Economic Review (ICER)
- China: National Healthcare Security Administration (NHSA)

Additional HTA bodies viewed as key in certain countries and otherwise influential in certain regions might include:

- Australia: Pharmaceutical Benefits Advisory Committee (PBAC)
- Brazil: National Committee for Technology Incorporation (CONITEC)
- Canada: Canada’s Drug Agency (CDA)
- Korea: National Evidence-based Healthcare Collaborating Agency (NECA)
- Netherlands: National Health Care Institute (ZIN)
- Scotland: Scottish Medicines Consortium (SMC)
- Sweden: Dental and Pharmaceutical Benefits Agency (TLV)
- Thailand: Health Intervention and Technology Assessment Program (HITAP)

Furthermore, the company will need to adapt its eHTA as needed, given the implementation of the European Union (EU) HTA Regulation during the interim period from 2025 to 2030. Among the regulation’s provisions are Joint Clinical Assessments for relative efficacy/effectiveness and safety, and the continuing evaluation of economic impacts and other nonclinical aspects by the designated HTA bodies of the individual EU nations. The regulation may improve the efficiency of technology development and evidence generation applicable across EU nations, although it will also affect the timeline and sequence of market access.

Consider that the emerging cancer therapy with a companion diagnostic noted above is being developed for certain cancers using a new mechanism of action. It is a “tumor agnostic” therapy that targets a specific biomarker (e.g., genetic mutation) that is a driver of tumor growth, regardless of the tumor’s anatomic origin. The company intends to conduct eHTA across multiple potential markets, each with national HTA bodies that influence access and reimbursement. The lines of inquiry for an eHTA of this cancer therapy across HTA bodies in the multiple target markets might include those listed in Table 2.

**Table 2.** Potential lines of eHTA inquiry to map the HTA terrain across multiple national markets

*Early advice:* What provisions are there for early regulatory and HTA advice?
*Evidence differences:* To what extent do the HTA body’s evidence requirements/expectations rely on and differ from those of the applicable regulatory agency?
*Clinical trial design:* What is acceptable for clinical trial design, e.g., RCT only, adaptive RCT, basket trial (to test how well the therapy works in patients with different types of cancer that share the same actionable biomarker), historical control, single arm trial, and other?
*Comparators:* For this type of therapy and target populations, what are acceptable comparators, e.g., standard of care, other active comparators, and indirect comparison (via common comparator across two or more trials)?
*Endpoints:* What are the preferred endpoints for therapies for these cancers, e.g., overall survival vs. one or more of progression-free survival, objective response rate, disease-free survival, therapy discontinuation, and other?
*Companion diagnostics:* What are evidence expectations for companion diagnostics (i.e., for detecting actionable biomarkers), e.g., for analytical and clinical validity, clinical utility, and impact on costs or cost-effectiveness?
*Real-world evidence:* What are the preferred or acceptable roles of real-world evidence, e.g., prospective or retrospective data, effectiveness in heterogeneous patient groups, long-term safety, treatment adherence/discontinuation, and expansion of indications?
*Health economics:* What types of health economic analysis are used, e.g., cost-effectiveness, cost-utility, cost–benefit, budget impact, reference pricing, and price negotiation? Are there preferred types of health economic modeling?
*Critical thresholds:* Do the findings/recommendations or price negotiations of this HTA body depend on an economic threshold or limit, e.g., a cost per quality-adjusted life year threshold or a maximum budget impact?
*Evidence adaptations:* To what extent are clinical evidence requirements or economic thresholds (if applicable) adaptable based on such factors as rare disease, unmet medical need, and disease severity?
*Other impacts:* What other types of impact or related considerations might affect HTA body findings/recommendations, e.g., health equity, patient and caregiver preferences for alternative benefit/risk profiles, and environmental impact?
*Managed access:* What are the provisions, if any, of managed access programs, such as managed entry agreements, coverage with evidence development, or risk-sharing agreements?
*Engagement:* What are the provisions for engagement in HTA by industry, health care providers, patients, and caregivers?

HTA, health technology assessment; RCT, randomized controlled trial.

## In summary

The article by Grutters et al. on the definition of eHTA and its explanatory narrative helps to recapture the originally intended broad scope of technology assessment and place it into a contemporary context of novel and potentially widely consequential health technologies.

As suggested by the examples above, eHTA may be especially advisable for technologies that pose potential challenges to usual practices in HTA. Anticipating how a novel type or class of health technology might fare in evolving regulatory and HTA frameworks should inform sponsors’ strategies for further development and opportunities for HTA bodies to plan for the arrival of such technologies on their dockets. Indeed, the largely favorable experience of how HTA continues to adapt its evidence expectations and related practices to new therapies for rare diseases is instructive. Today, this might apply to, for example, various AI-enabled health technologies, such as circulating tumor DNA for screening, early detection, use as companion diagnostics, or monitoring; tumor-agnostic cancer therapies; prescription digital therapeutics; and various applications of remote patient monitoring. At a higher level, eHTA could take on multi-adaptable “platform technologies” in health care, such as messenger RNA vaccine development, computer-aided drug design, digital health platforms, and AI foundation models.

The new definition of eHTA reflects a long-held recognition in HTA of the need to stay ahead of the curve in terms of both intended and unintended impacts of health technologies. As one health technology assessor observed in 1987 about the tradeoffs of early assessment with limited evidence versus later assessment after a technology has gotten a foothold in a healthcare system:

It’s always too early until, unfortunately, it’s suddenly too late! (4).
