# Defining early health technology assessment: reclaiming origins and diversity of applications – ERRATUM

**DOI:** 10.1017/S0266462325100305

**Published:** 2025-07-28

**Authors:** Clifford Goodman

The above manuscript has been published with an error in a sentence in paragraph 2 of the summary, the sentence reads:

Today, this might apply to, for example, various AI-enabled health technologies, such as circulating tumor DNA for screening, early detection, use as companion diagnostics, or monitoring; tumoragnostic cancer therapies; prescription digital therapeutics; and various applications of remote patient monitoring.

The sentence should read:

Today, this might apply to, for example, various AI-enabled health technologies; circulating tumor DNA for screening, early detection, use as companion diagnostics, or monitoring; tumor-agnostic cancer therapies; prescription digital therapeutics; and various applications of remote patient monitoring.

The publisher apologizes for this error.
